# Supplementary material for: RNA polymerase II primes Polycomb‐repressed developmental genes throughout terminal neuronal differentiation
Source: Mol Syst Biol. 2017 Oct 16;13(10):946. doi: 10.15252/msb.20177754 (PMC5658700; doi:10.15252/msb.20177754)
Supplement: Supplementary file 1 — Appendix [file MSB-13-946-s001.pdf]

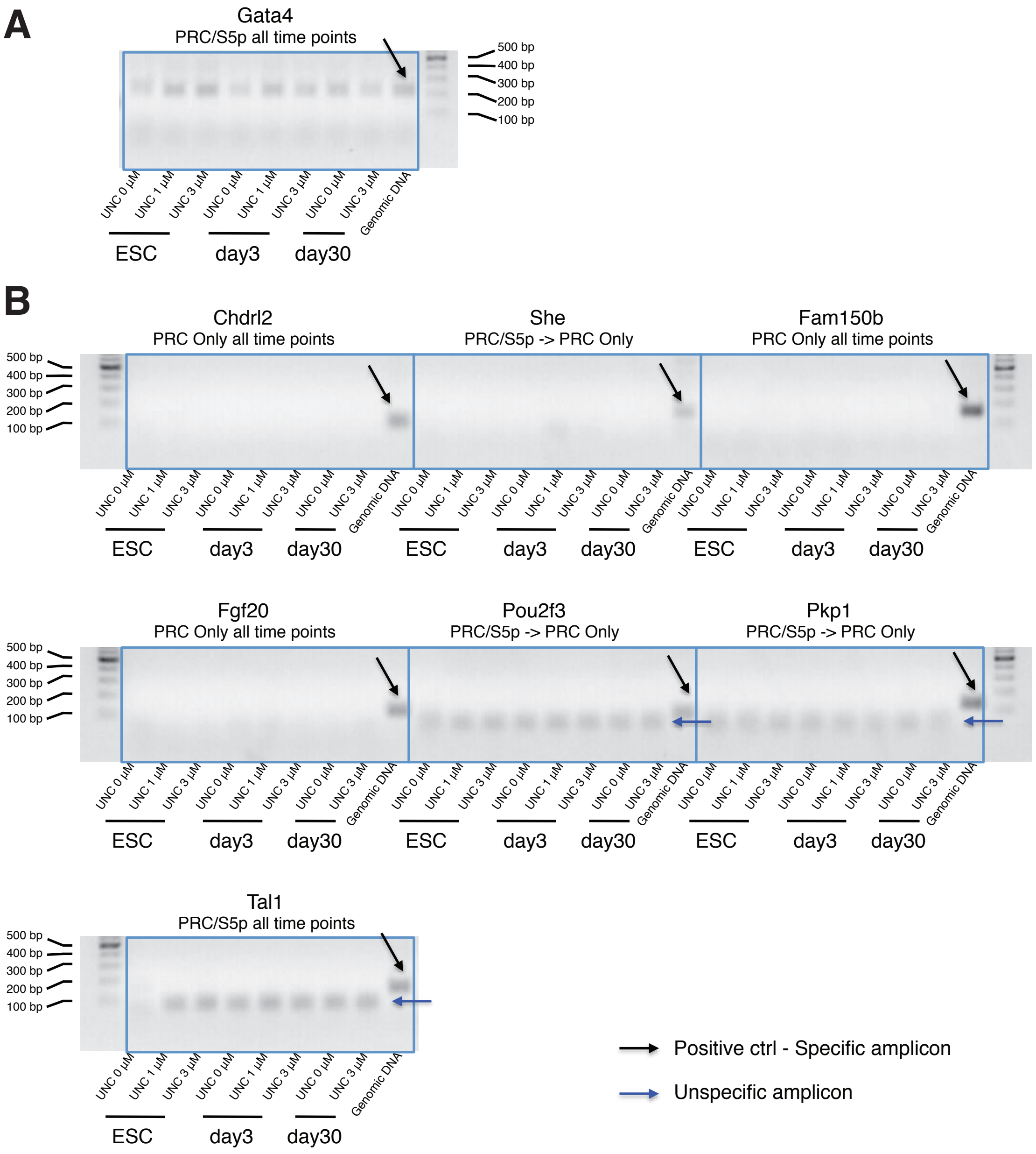

Appendix Figure S1. Quality of PCR primers was verified by agarose gel electrophoresis.
qRT-PCRs were performed using total RNA as reported in Materials and Methods for genes classified as PRC/S5p at all time points, PRC/S5p -> PRC Only, and PRC Only at all time points. Genomic DNA was amplified in parallel as a positive control to control for primer amplification. PCR products were analyzed on 2% (w/v) agarose gels in 1xTAE buffer.
PCRs products from samples treated with different concentrations of the PRC2 inhibitor UNC1999 are compared with a PCR positive control.
(A) Gata4 RNA amplification shows amplification of the specific amplicon.
(B) Genes for which the correct amplicons were not detected in total RNA, but confirmed in genomic DNA.
