# Supplementary material for: RNA polymerase II primes Polycomb‐repressed developmental genes throughout terminal neuronal differentiation
Source: Mol Syst Biol. 2017 Oct 16;13(10):946. doi: 10.15252/msb.20177754 (PMC5658700; doi:10.15252/msb.20177754)
Supplement: Supplementary file 2 — Expanded View Figures PDF [file MSB-13-946-s002.pdf]

Expanded View Figures

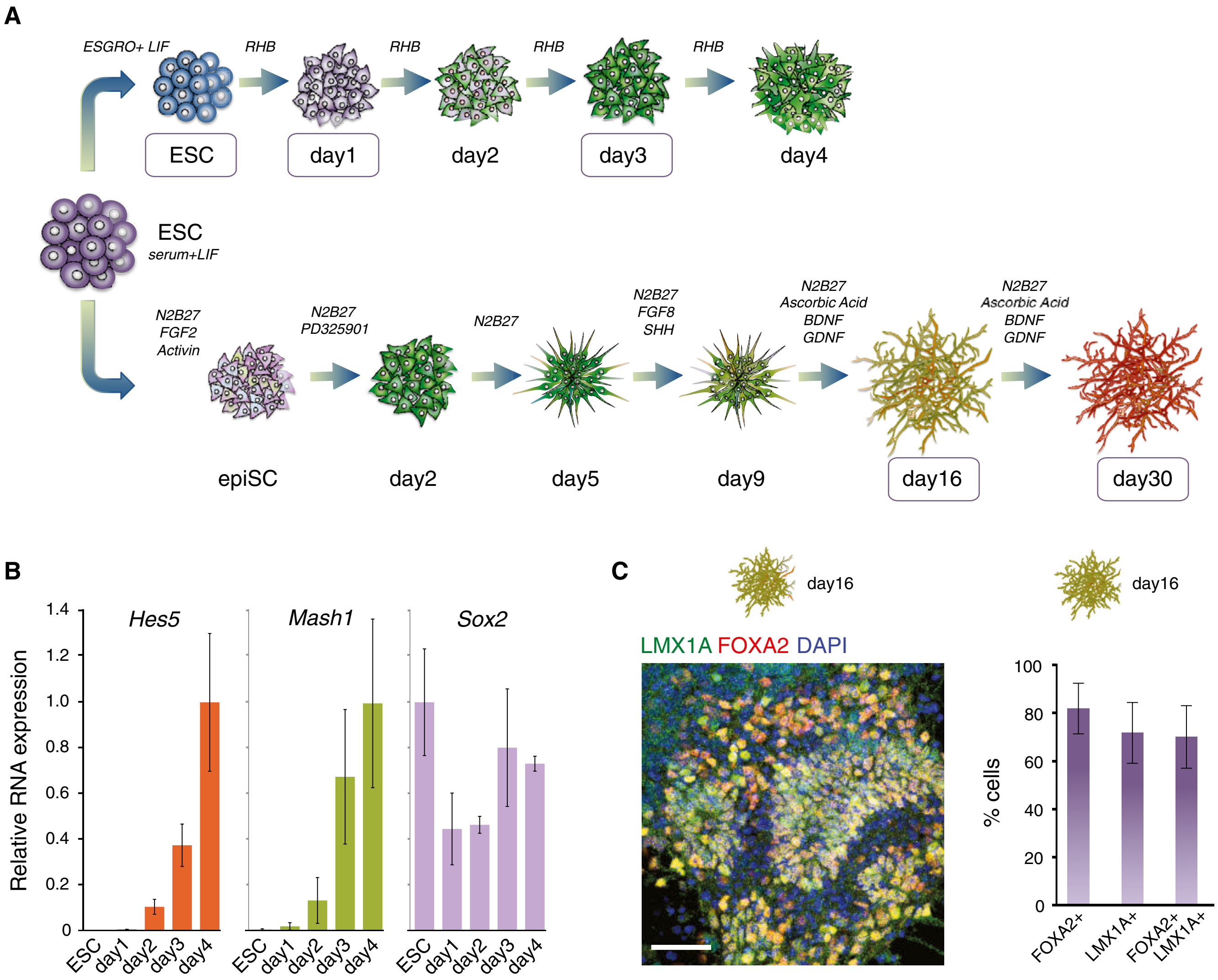

Figure EV1. Neuronal differentiation protocol starting from ESCs gives cultures enriched for ventral midbrain dopaminergic neurons.
Related to Fig 1.

A Schematic representation of the protocols used to differentiate ESCs to dopaminergic neurons. Time points selected for ChIP-seq and mRNA-seq are boxed.

B Total RNA qRT–PCR shows the differential expression of specific markers during exit from pluripotency. Relative levels are normalized to *Actb* and plotted as ratio to the expression in the most expressed time point. Mean and standard deviation (SD) are from three biological replicates.

C Left, indirect immunofluorescence of LMX1A (green) and FOXA2 (red) in day 16 neurons. Nuclei were counterstained with DAPI (blue). Scale bar, 100 µm. Right, percentage of cells positive for FOXA2, LMX1A, and both. Mean and SD are from five fields of view.

 © 2017 The Authors

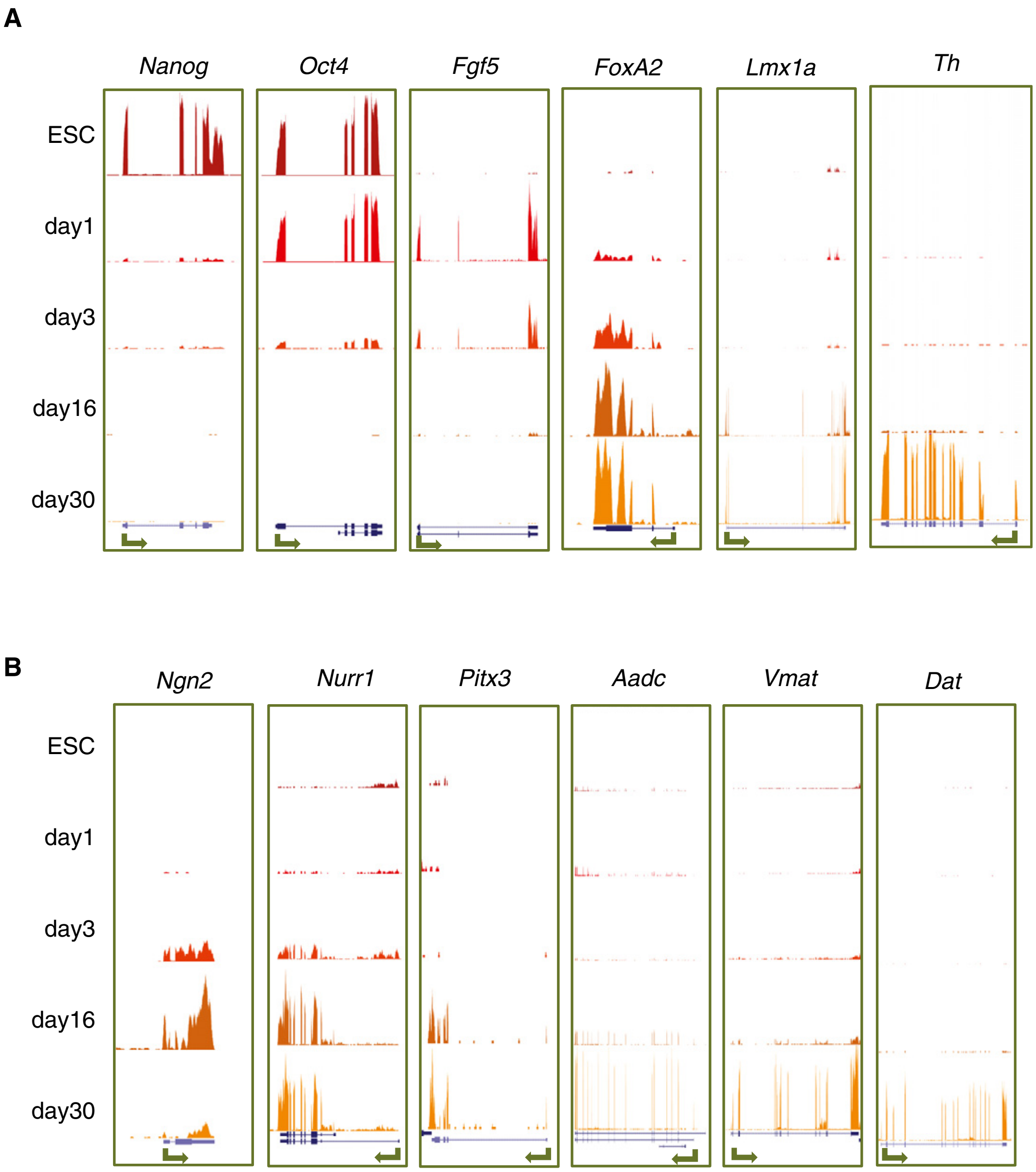

Figure EV2. mRNA-seq profiles highlight single-gene expression changes at different stages of differentiation.
Related to Fig 1.

A UCSC Genome Browser snapshots of mRNA-seq tracks confirm the expression of specific markers for differentiation. ESCs progressively downregulate pluripotency genes, such as *Nanog* and *Oct4*, and express *Fgf5* at the onset of differentiation. *FoxA2* and *Lmx1a* are expressed in developing neurons while *Th* in mature dopaminergic neurons.

B UCSC Genome Browser snapshots of mRNA-seq tracks for additional markers of developing (*Ngn2*, *Nurr1*, *Pitx3*) and mature (*Aadc*, *Vmat*, *Dat*) dopaminergic neurons are sequentially activated in terminally differentiated neurons.

Data information: Arrows represent the position of promoter and directionality of the gene. *Y*-axis scales are kept constant per gene and adjusted to its maximum expression.

Figure EV3. Functional and phenotypical characterization of neurons during maturation from day 14 to day 30.
Related to Fig 2.

A Spontaneous action potential firing frequency and regularity across differentiation days. Graphs show mean and standard error of the mean (SEM) of frequency and regularity of action potential activity across differentiation (n = 3, 8, 4, 20; Frequency ANOVA = 4.371, P-value = 0.2241; CV-ISI ANOVA = 8.007, P-value = 0.0459). Coefficient of variation of the inter-spike-interval (CV-ISI) decreases, reflecting an increase in regularity.

B Example trace of spontaneous bursting in a mature neuron indicated by the asterisk. In the lower panel, the burst is displayed over a smaller time scale to better observe the burst properties (i.e., decline in action potential amplitude within the burst).

C Burst firing was only observed after 30 days in culture. Percentage of spikes per burst and the number of spikes per burst across differentiation (n = 3, 8, 5, 20; chi-squared test for trend = 5.57, P-value = 0.0183).

D Example trace showing spontaneous action potential firing blocked by 1 μM tetrodotoxin (TTX).

E Example traces of miniature excitatory post-synaptic currents (mEPSCs) observed in the presence of 1 μM TTX. mEPSCs were blocked with 5 μM NBQX, confirming their glutamatergic identity.

F Example traces of miniature inhibitory post-synaptic currents (mIPSCs) observed in the presence of 1 μM TTX in the external solution and high Cl in the recording pipette. mIPSCs were blocked with 100 μM picrotoxin, confirming their GABAergic identity.

G Confocal images showing the neuronal subtypes that are present within the cultures. Indirect immunofluorescence of TH (green) denotes dopaminergic neurons, VGluT2 (magenta) denotes glutamatergic neurons, and GABA (blue) denotes GABAergic neurons, with the overlay of all channels in the bottom right panel. Scale bars, 30 μm.

H UCSC Genome Browser snapshots show the expression of GABAergic and glutamatergic neuronal markers. GABAergic and glutamatergic neurons are important to establish neuronal circuits and ultimately to obtain functional dopaminergic neurons. Arrows represent the position of promoter and directionality of the gene. *Y*-axis scales are kept constant per gene and adjusted to its maximum expression.

I Mature neurons do not form autapses. Example trace shows a short current pulse that evoked an action potential, but no rebound post-synaptic depolarization.

© 2017 The Authors

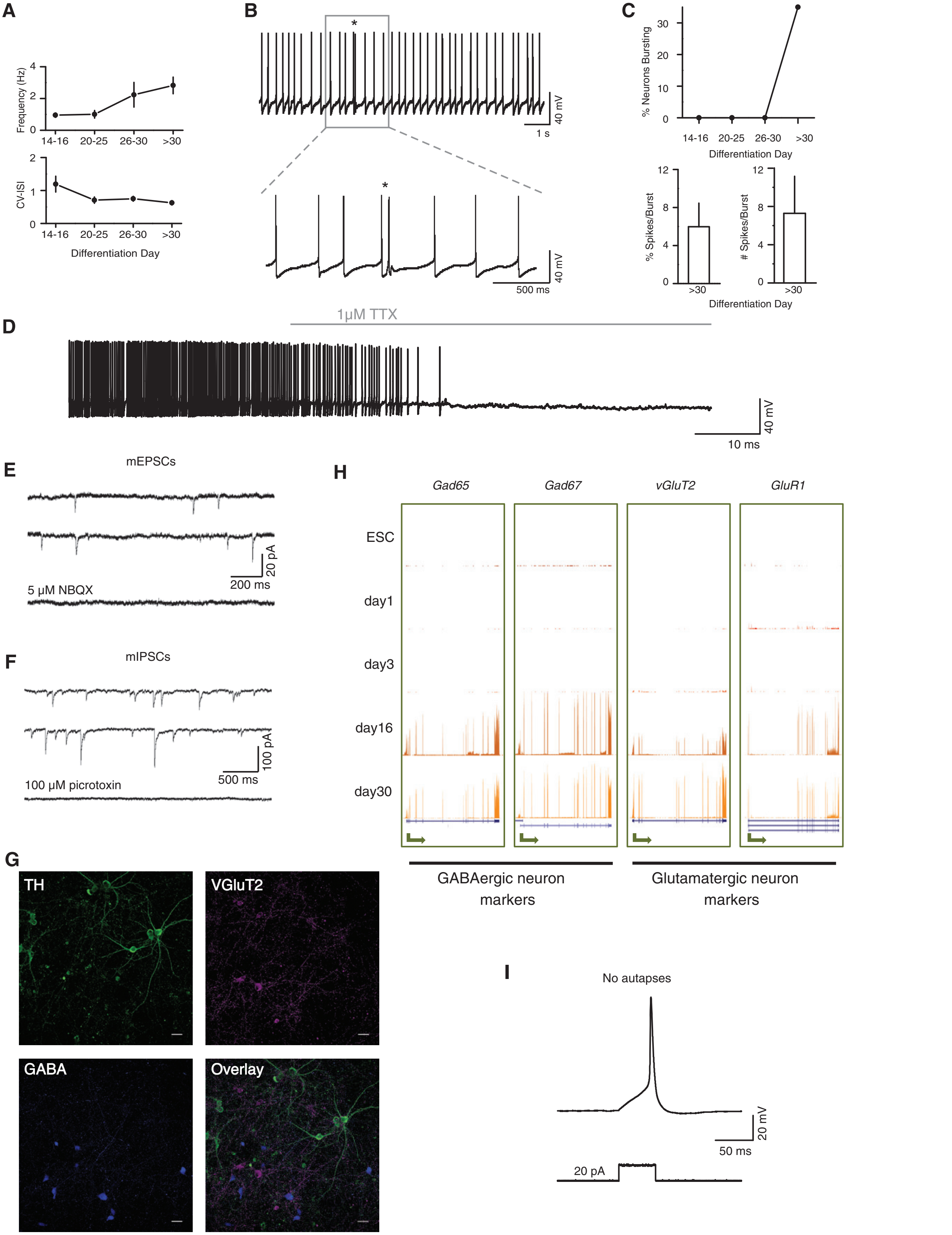
A
Frequency (Hz)
CV-ISI
14-16
20-25
26-30
>30
Differentiation Day
B
40 mV
1 s
500 ms
C
% Neurons Bursting
% Spikes/Burst
# Spikes/Burst
D
1μM TTX
10 ms
E
mEPSCs
5 μM NBQX
20 pA
200 ms
F
mIPSCs
100 μM picrotoxin
100 pA
500 ms
G
TH
VGluT2
GABA
Overlay
H
Gad65
Gad67
vGluT2
GluR1
ESC
day1
day3
day16
day30
GABAergic neuron markers
Glutamatergic neuron markers
I
No autapses
20 mV
50 ms
20 pA

Figure EV3.

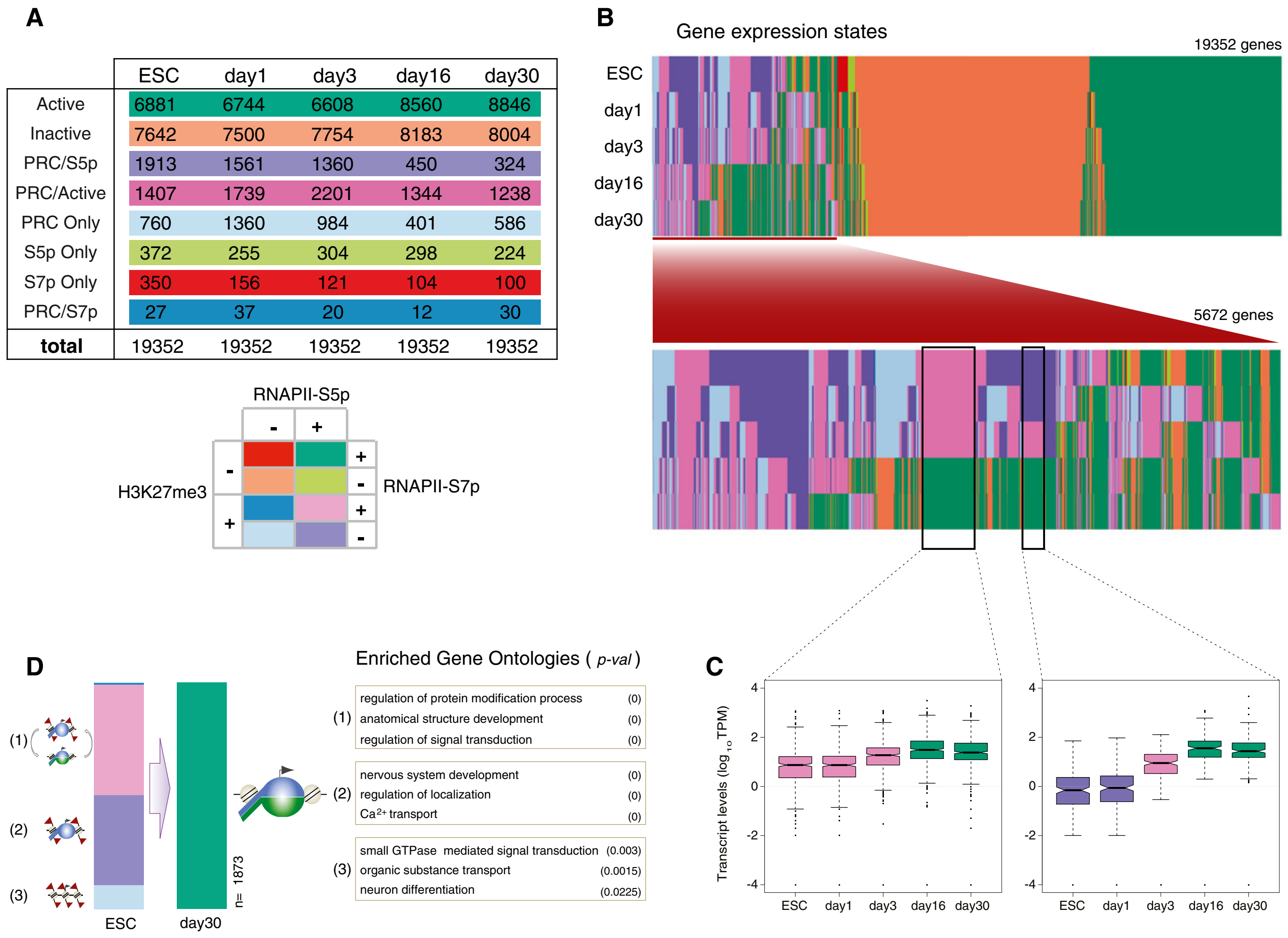

	ESC	day1	day3	day16	day30
Active	6881	6744	6608	8560	8846
Inactive	7642	7500	7754	8183	8004
PRC/S5p	1913	1561	1360	450	324
PRC/Active	1407	1739	2201	1344	1238
PRC Only	760	1360	984	401	586
S5p Only	372	255	304	298	224
S7p Only	350	156	121	104	100
PRC/S7p	27	37	20	12	30
**total**	19352	19352	19352	19352	19352

Figure EV4. Gene classification based on promoter association with PRC and RNAPII at different stages of the neuronal differentiation.
Related to Fig 5.

A Number of genes in each promoter state described in Fig 5A. Classification based on the presence or absence of RNAPII-S5p, RNAPII-S7p, and H3K27me3 at the 2-kb window centered around promoters, shown from ESC to day 30 neurons.

B Promoter state dynamics during differentiation for all classified genes. Bottom panel shows the promoter state dynamics for genes that are marked by H3K27me3 in at least one time point.

C Gene expression changes for two selected groups of genes that become Active from Polycomb-repressed states (highlighted by black boxes in panel B). Colors indicate promoter states in each time point. In boxplots, as default of R graphics package, box center represents the median value; box top and bottom represent third and first quartiles, respectively. Notches extend to $\pm$ 1.58 $\times$ Interquartile range (IQR)/n. Whiskers extend to the highest point within 1.5 IQR of the third quartile and to the lowest point within 1.5 IQR of the first quartile, respectively. Values outside whiskers are represented as individual points.

D Promoter state of genes that are marked by PRC in ESCs and become Active in day 30 neurons. Examples of enriched GO terms are shown using as background all genes. Permute *P*-value (GO-Elite) is shown.

 © 2017 The Authors

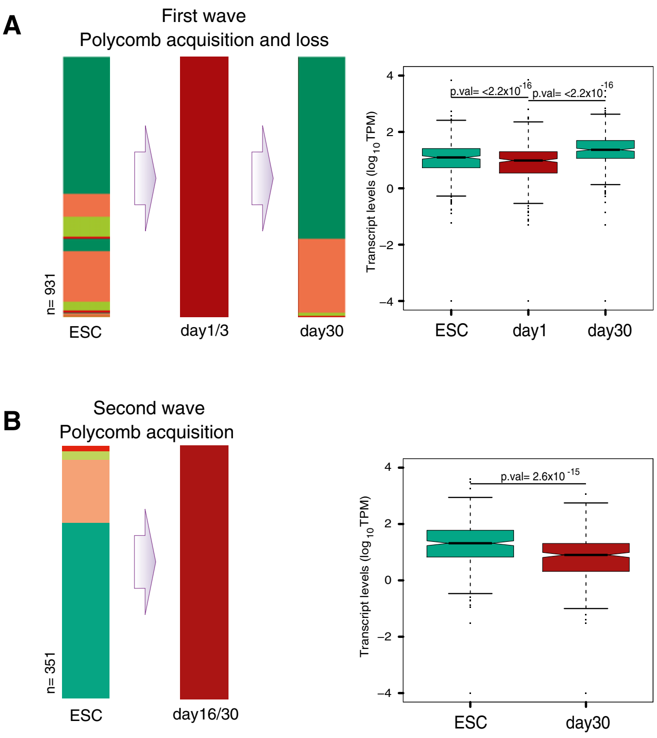

Figure EV5. Promoter state changes involving PRC loss or acquisition in intermediate or late stages of neuronal differentiation.
Related to Fig 5.

A Most genes that acquire H3K27me3 *de novo* during the early phases of differentiation (days 1 or 3) have Active promoters in ESCs and in day 30 neurons. Acquisition of PRC by genes with Active promoters in ESCs is accompanied by a decrease of gene expression, as exemplified for genes that acquire H3K27me3 at day 1 and become Active again in day 30 (n = 358). P-values were calculated using Wilcoxon signed rank (paired) test.

B Genes that acquire H3K27me3 *de novo* in day 16 or 30 neurons tend to have Active promoters in ESCs. Active genes in ESCs that gain PRC in day 30 show decreased mRNA expression (n = 311). P-values were calculated using Wilcoxon signed rank (paired) test.

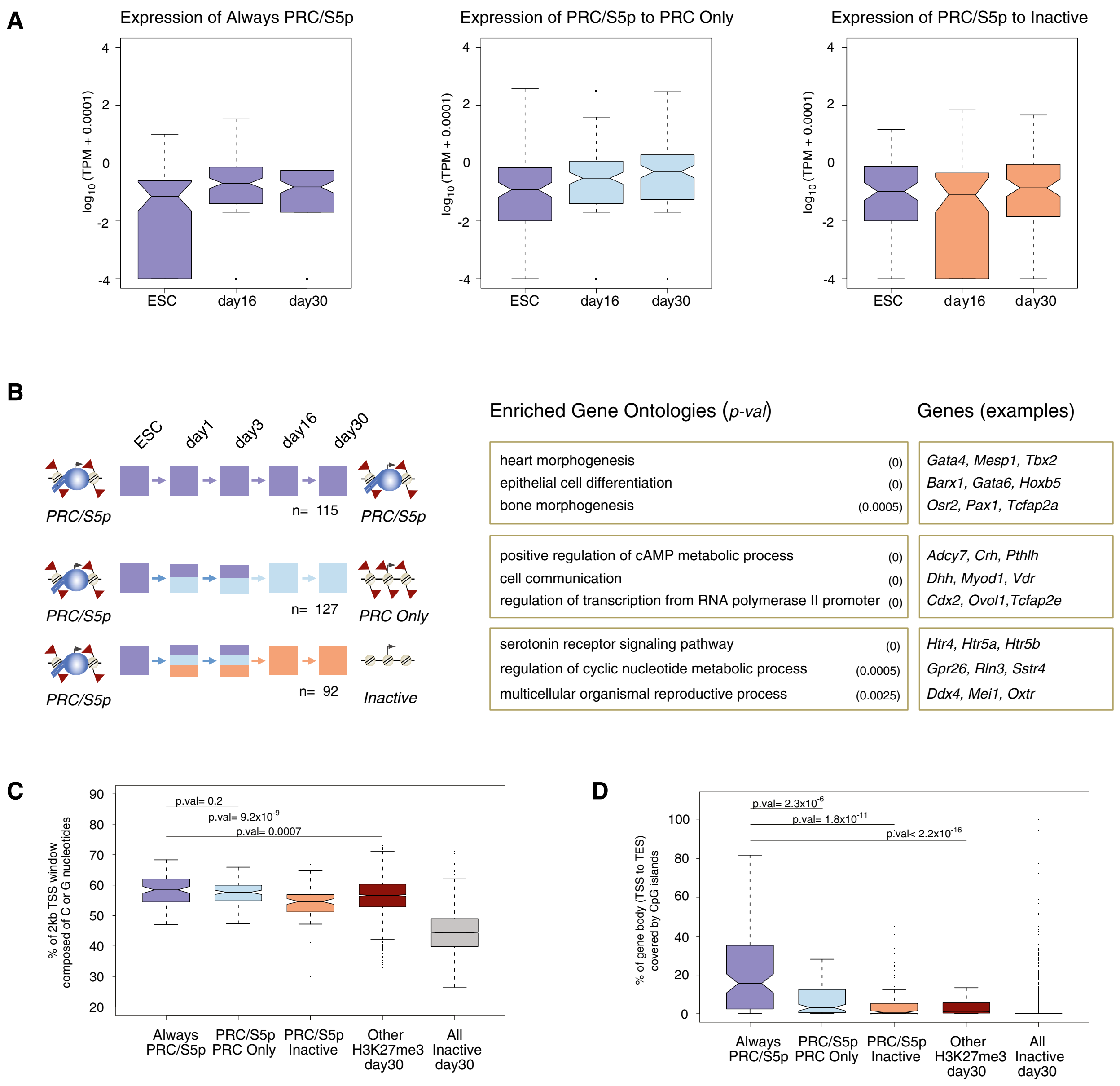

Figure EV6. Genes that maintain the PRC/S5p promoter state throughout differentiation are often morphogenic TFs linked to different tissue identities and show high coverage by CpG islands.

Related to Fig 7.

- A Similar low levels of mRNA expression are detected in ESC, day 16, and day 30 neurons for genes that are PRC/S5p in ESCs and remain silent through differentiation either by retaining the PRC/S5p promoter state, or by becoming PRC Only or Inactive. Colors indicate gene groups according to panel (B).
- B Examples of enriched Gene Ontologies and associated genes of the three groups described in panel (A), using as background all genes. PRC/S5p genes that retain the PRC/S5p state throughout differentiation include important morphogenetic TFs for different cell lineages. Permute *P*-value (GO-Elite) is shown.
- C G/C composition of promoter windows for different groups of genes. Always PRC/S5p promoters are more enriched in G/C nucleotides compared to PRC/S5p genes that resolve to Inactive, or to the group of additional genes marked by H3K27me3 in day 30. Inactive genes in day 30 are shown for comparison. *P*-values were calculated using Wilcoxon rank-sum test.
- D Overlap of CpG islands with gene bodies for different groups of genes. The gene bodies of Always PRC/S5p are significantly more covered with CpG islands than PRC/S5p genes that become PRC Only or Inactive, or than the group of other $H3K27me3^{+}$ genes in day 30. Inactive genes in day 30 are shown for comparison. *P*-values were calculated using Wilcoxon rank-sum test.

 © 2017 The Authors

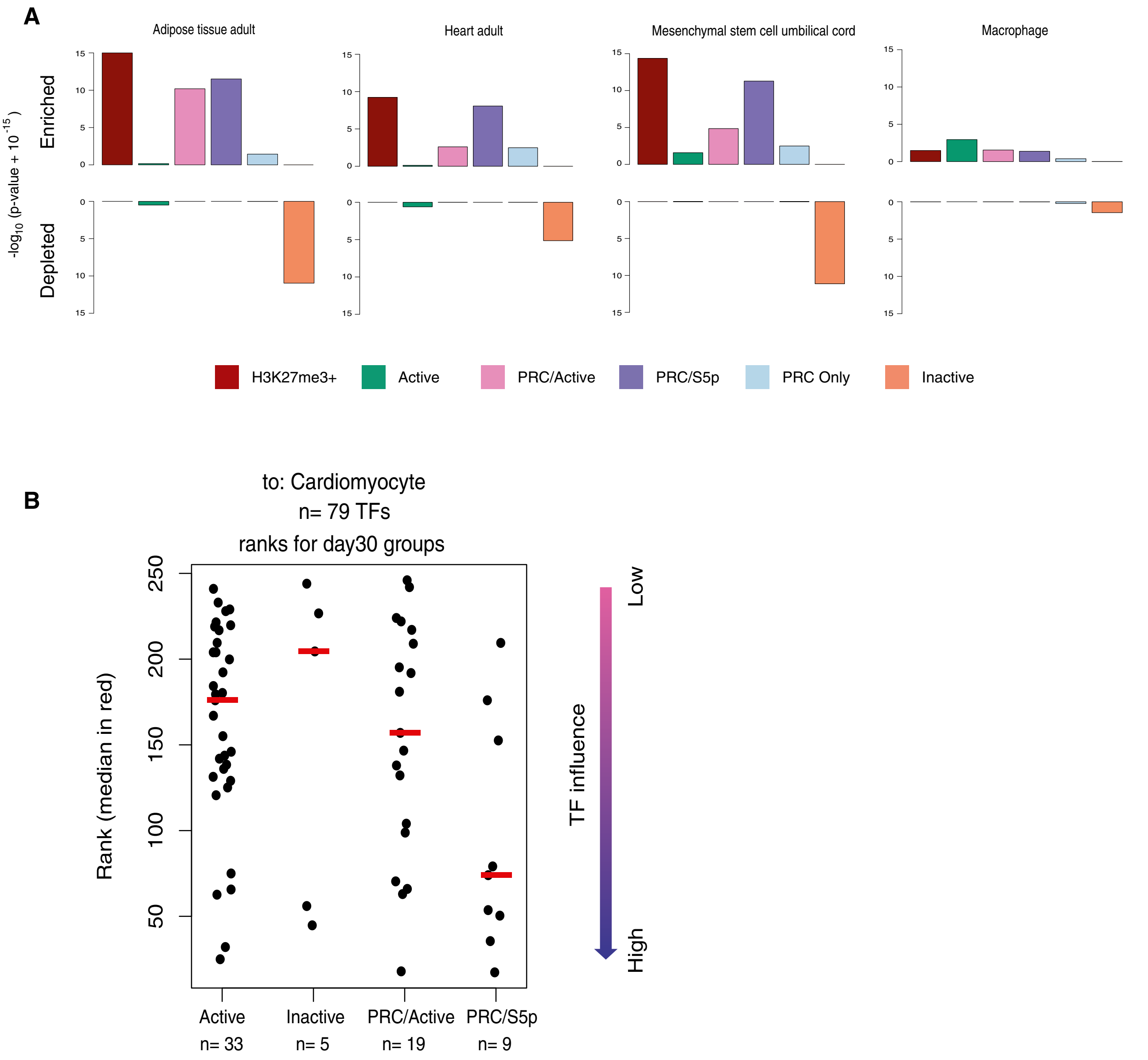

Figure EV7. Candidate genes with high influence for trans-differentiation are associated with PRC⁺ promoters in dopaminergic neurons.
Related to Fig 8.

A Additional examples of day 30 promoter state enrichment in Mogrify lists of candidate genes predicted to influence trans-differentiation toward different cell types. Different classes of PRC^+ promoters are enriched, whereas genes with Inactive promoters are depleted. Enrichment and depletion *P*-values were calculated with the hypergeometric test.

B Mogrify identifies both the TFs with high influence to trigger change from cell type A to cell type B and the rank order according to their predicted influence in the conversion. Example showing that PRC/S5p and PRC/Active genes are predicted to have higher influence in trans-differentiation toward cardiomyocytes compared to Active or Inactive genes. Median Mogrify rank is shown in red.

© 2017 The Authors
